# Supplementary material for: Generation of HIV-Resistant Macrophages from IPSCs by Using Transcriptional Gene Silencing and Promoter-Targeted RNA
Source: Mol Ther Nucleic Acids. 2018 Aug 4;12:793–804. doi: 10.1016/j.omtn.2018.07.017 (PMC6111070; doi:10.1016/j.omtn.2018.07.017)

OMTN, Volume *12*

Supplemental Information

Generation of HIV-Resistant Macrophages from IPSCs by Using Transcriptional Gene Silencing and Promoter-Targeted RNA

Kei Higaki, Masako Hirao, Ai Kawana-Tachikawa, Shoichi Iriguchi, Ayako Kumagai, Norihiro Ueda, Wang Bo, Sanae Kamibayashi, Akira Watanabe, Hiromitsu Nakauchi, Kazuo Suzuki, and Shin Kaneko

Figure S1. Karyotype analysis of iPSCs transduced with shRNA.

(a) Karyotyping of sh-transduced iPSC: 95% (19/20) of PromA-transduced and 100% (20/20) of M2-transduced iPSC showed normal karyotype.

(b) X-chromozome FISH assay of PromA-iPSC after sub-cloning: After simple sub-cloning, 99.7% of 1000 PromA-iPSCs showed normal X-choromosomal signal as same as healthy donor PBMC.

Figure S2. PCR analysis of iPSCs transduced with shRNA. (a) Ethidium bromide-stained agarose gels of the products of PCRs performed using shRNA-specific primers, which yielded amplicons of the predicted size in iPSCs transduced with shPromA (PromA-M2) and shPromA-M2 (M2). (b) Ethidium bromide-stained agarose gels of the products of PCRs performed using shRNA-specific primers, which yielded amplicons of the predicted size in macrophages transduced with shPromA (PromA) and shPromA-M2 (M2), but not in macrophages derived from untransduced iPSCs (WT). (c) PCR primer position used for the confirmation of the presence of transgene in the iPSCs.

Figure S3. Flow cytometric analysis of CD4 on iPSC-derived macrophages. Macrophages derived from iPSCs transduced with shPromA or shPromA-M2, and control

iPSCs (PromA, M2, and WT respectively) were analyzed for CD4 expression (black). Unstained macrophages derived from wild-type iPSCs are shown as negative controls (white).

FigureS1

a. Karyotyping of sh-transduced iPSC

G-banding	PromA-iPSC	M2-iPSC
46XX	19/20 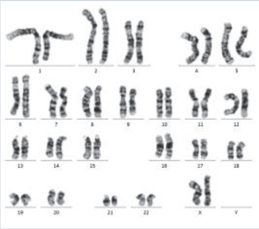	20/20 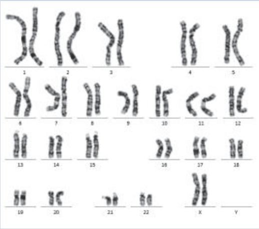
47XXX	1/20 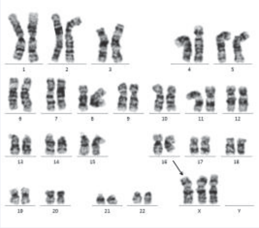	0/20

b. X-chromosome FISH assay of PromA-iPSC after sub-cloning

Signal number by DXZ1(Xcen) probe	PromA-iPSC Cell # (% in 1000 cells)	Healthy donors PBMC Cell # (% in 1000 cells)
1	5 (0.5%)	10 (1.0%)
2	993 (99.3%)	985 (98.5%)
3	2 (0.2%)	5 (0.5%)

FigureS2

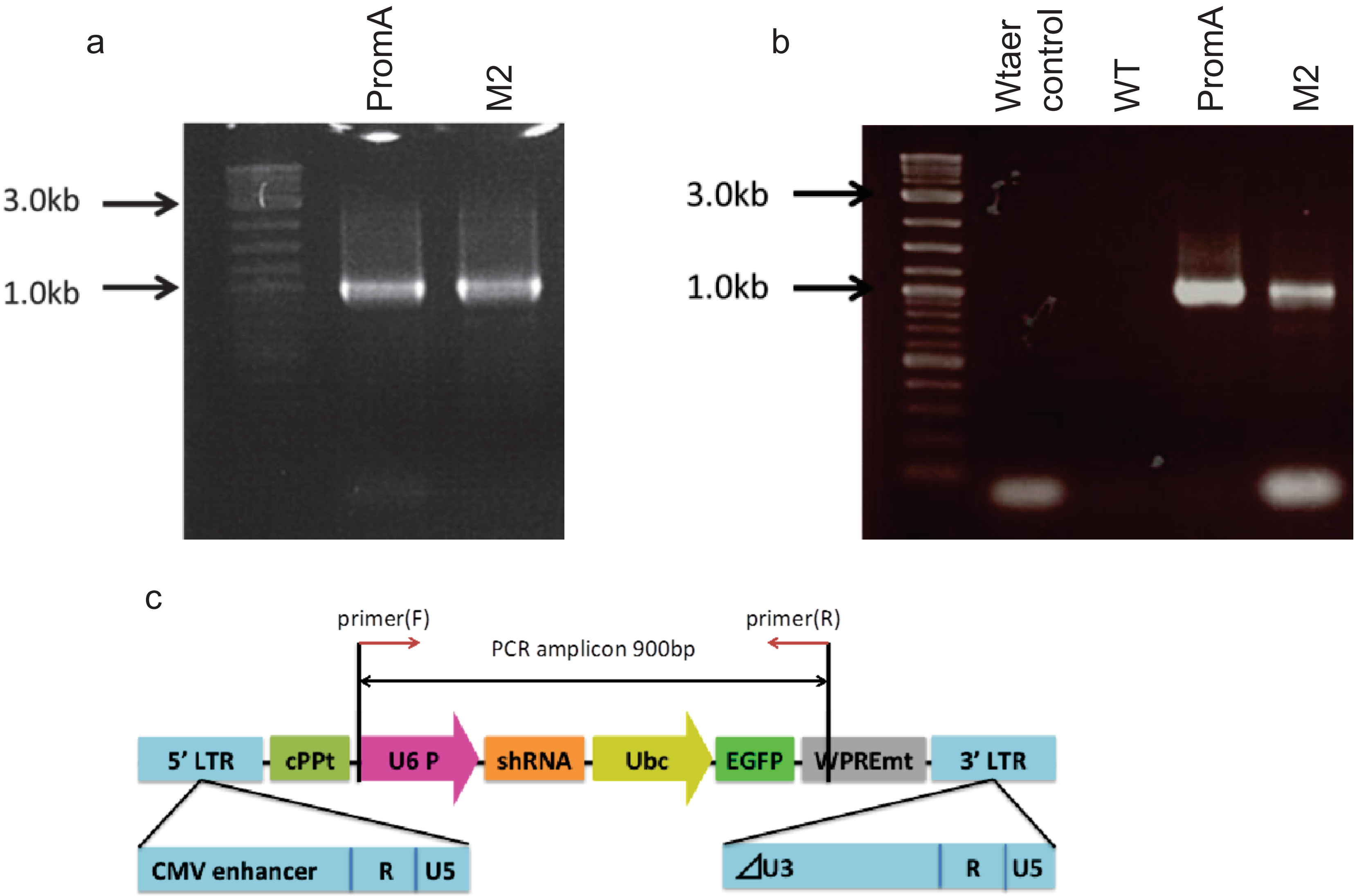

forward primer	5’-CACCGAGGGCCTATTTCCCATGA-3’
reverse primer	5’-ACCTTGTTTCAACGACCTCACAGC-3’

FigureS3

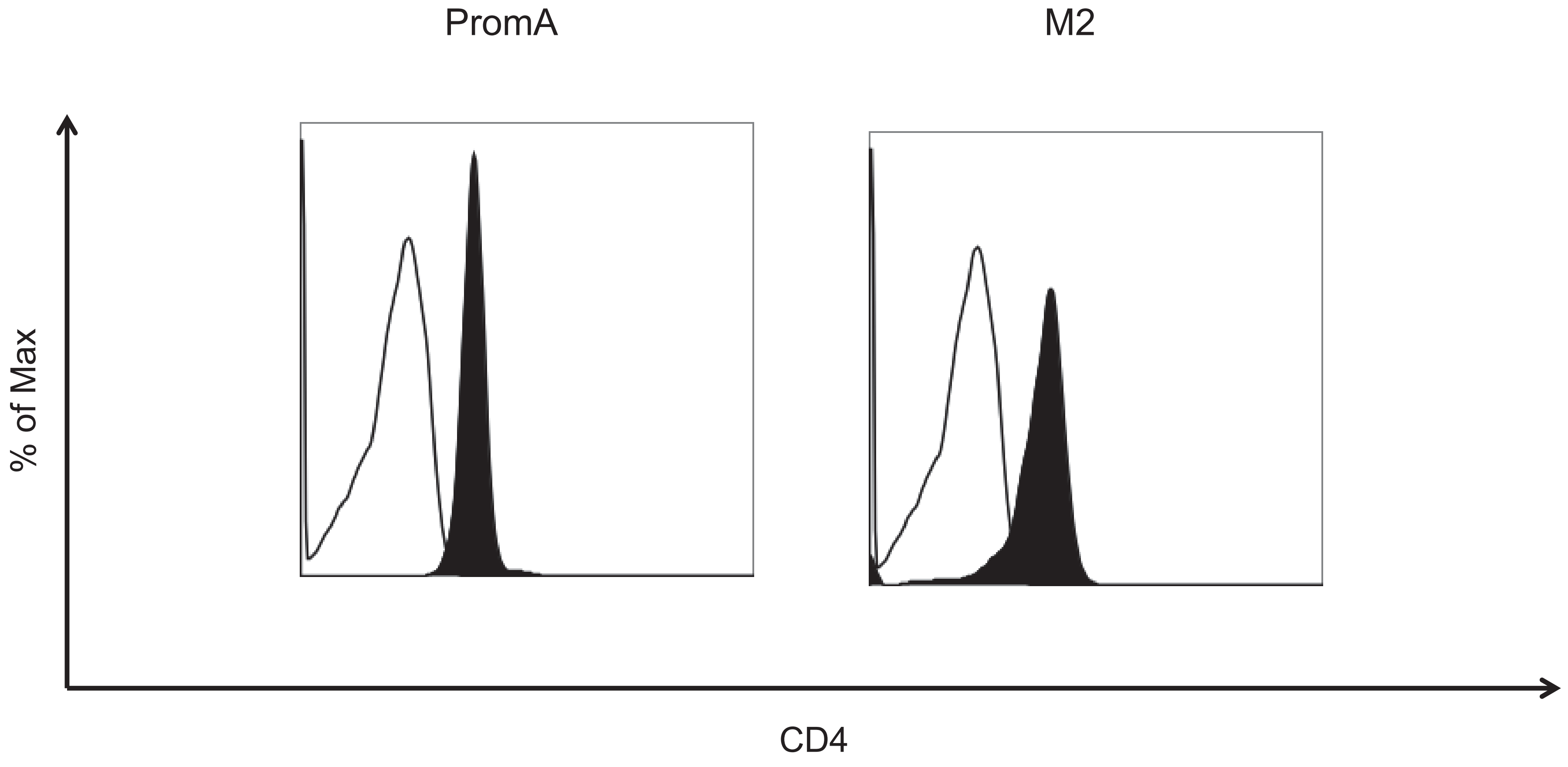

Supplement: Document S1. Figures S1–S3 [file mmc1.pdf]
