# Supplementary material for: Generation of HIV-Resistant Macrophages from IPSCs by Using Transcriptional Gene Silencing and Promoter-Targeted RNA
Source: Mol Ther Nucleic Acids. 2018 Aug 4;12:793–804. doi: 10.1016/j.omtn.2018.07.017 (PMC6111070; doi:10.1016/j.omtn.2018.07.017)

Molecular Therapy
Nucleic Acids
Original Article

Generation of HIV-Resistant Macrophages from IPSCs by Using Transcriptional Gene Silencing and Promoter-Targeted RNA

Kei Higaki,[1,7] Masako Hirao,[1,7] Ai Kawana-Tachikawa,[2] Shoichi Iriguchi,[1] Ayako Kumagai,[1] Norihiro Ueda,[1] Wang Bo,[1] Sanae Kamibayashi,[1] Akira Watanabe,[3] Hiromitsu Nakauchi,[4,5] Kazuo Suzuki,[6] and Shin Kaneko[1]

[1]Shin Kaneko Laboratory, Department of Cell Growth and Differentiation, Center for iPS Cell Research and Application (CiRA), Kyoto University, Sakyo-ku, Kyoto 606-8501, Japan; [2]AIDS Research Center, National Institute of Infectious Diseases, Shinjuku-ku, Tokyo 162-8640, Japan; [3]Watanabe Laboratory, Department of Life Science Frontier, Center for iPS Cell Research and Application (CiRA), Kyoto University, Shogoin, Sakyo-ku, 606-8501 Kyoto, Japan; [4]Division of Stem Cell Therapy, Institute of Medical Science, University of Tokyo, Tokyo, Japan; [5]Institute for Stem Cell Biology and Regenerative Medicine, Stanford University School of Medicine, Stanford, CA 94305, USA; [6]St Vincent's Centre for Applied Medical Research (AMR), St Vincent's Hospital, Darlinghurst, NSW 2010, Australia

Highly active antiretroviral therapy (HAART) has markedly prolonged the prognosis of HIV-1 patients. However, lifelong dependency on HAART is a continuing challenge, and an effective therapeutic is much desired. Recently, introduction of short hairpin RNA (shRNA) targeting the HIV-1 promoter was found to suppress HIV-1 replication via transcriptional gene silencing (TGS). The technology is expected to be applied with hemato-lymphopoietic cell transplantation of HIV patients to suppress HIV transcription in transplanted hemato-lymphopoietic cells. Combination of the TGS technology with new cell transplantation strategy with induced pluripotent stem cell (iPSC)-derived hemato-lymphopoietic cells might contribute to new gene therapy in the HIV field. In this study, we evaluated iPSC-derived macrophage functions and feasibility of TGS technology in macrophages. Human iPSCs were transduced with shRNAs targeting the HIV-1 promoter region (shPromA) by using a lentiviral vector. The shPromA-transfected iPSCs were successfully differentiated into functional macrophages, and they exhibited strong protection against HIV-1 replication with alteration in the histone structure of the HIV-1 promoter region to induce heterochromatin formation. These results indicated that iPS-derived macrophage is a useful tool to investigate HIV infection and protection, and that the TGS technology targeting the HIV promoter is a potential candidate of new gene therapy.

INTRODUCTION

The currently available combined antiviral therapy prevents the occurrence of symptoms related to AIDS associated with HIV-1 infection.[1–3] However, these highly active antiretroviral therapies (HAARTs) do not impact the viral reservoirs where HIV-1 persists in its proviral form, and their cessation leads to rapid viral recurrence except in a few cases.[4,5] In addition, previous studies showed the emergence of a multiple drug-resistant strain of HIV-1 and a range of adverse effects from the drug-based therapy, which appear to be cumulative.[6,7] Therefore, alternative therapeutic approaches for HIV-1 infection are required, and one of the potent candidates is gene therapy.

Inhibition of HIV-1 through post-transcription gene silencing (PTGS) has been reported previously.[8–12] Small interfering RNAs (siRNAs) and short hairpin RNA (shRNA) of about 22 nucleotides targeting HIV-1 structural and accessory genes induce rapid degradation of mRNA containing complementary sequence and suppress the production of new virus *in vitro*. The duration of this effect varies from 4 to 7 days.[8] However, prolongation of this effect has been achieved using adeno-associated virus or lentiviral vectors to deliver stably expressed shRNA. HIV-1 can be suppressed for between 14 and 25 days by using this approach. HIV-1 is known to adapt to environmental pressures, and rapid selection of shRNA escape mutants has been described *in vitro*.[13,14]

We sought an alternative approach of gene therapy to inhibit rapid viral adaptation to environmental pressures and to suppress viral replication for extended periods. Transcriptional gene silencing (TGS) mediated by siRNAs targeting promoter regions of genes of interest was first described in plants.[15,16] Previous studies have shown that siRNAs targeting the promoter region potently suppress HIV-1 via TGS in mammalian cells.[17,18] Another study found the suppressive effect of shRNA-mediated TGS on HIV-1 in primary monocyte-derived macrophages (MDMs).[19] These studies suggest that the shRNA-mediated TGS approach to suppress viral replication is

Received 2 December 2017; accepted 27 July 2018;
https://doi.org/10.1016/j.omtn.2018.07.017.

[7]These authors contributed equally to this work.

Correspondence: Shin Kaneko, Shin Kaneko Laboratory, Department of Cell Growth and Differentiation, Center for iPS Cell Research and Application (CiRA), Kyoto University, 53 Shogoin Kawahara-cho, Sakyo-ku, Kyoto 606-8501, Japan.
E-mail: kaneko.shin@cira.kyoto-u.ac.jp
Correspondence: Kazuo Suzuki, St Vincent's Centre for Applied Medical Research (AMR), St Vincent's Hospital, Darlinghurst, NSW 2010, Australia.
E-mail: k.suzuki@amr.org.au

This is an open access article under the CC BY license (http://creativecommons.org/licenses/by/4.0/).

applicable to a wide range of cell types. We reported previously that the TGS approach leads to persistence of anti-HIV effect for as long as 1 year without developing any evidence of escape-resistant mutation.[20] This is because siRNA- and shRNA-mediated TGS are associated with the induction of epigenetic changes in the HIV promoter, which are associated with the suppression of HIV-1 transcription.[21–23]

The viral reservoir in long-lived T cells and myeloid lineage cells plays an important role in viral recurrence in the absence of HAART.[24] A recent study also focused on permanently locking the reservoir in a silent state by using the TGS approach.[19] Resistance to reactivation of latent HIV-1 infection was observed in HIV-1 J-Lat 9.2 cells transduced with promoter-targeted shRNA.[19] Thus, the TGS approach can provide a means to overcome the current barrier to block HIV-1 transcription in the latently infected reservoir. Recent studies have shown that TGS targeting the HIV-1 promoter region induced marked reduction of HIV-1 transcription by altering the histone methylation and DNA methylation status within the HIV-1 promoter,[20–22] which is characteristic of facultative heterochromatin. However, off-target activity was also observed in siRNAs designed to induce TGS.[25–27] A previous study showed that some siRNA sequences stimulate an immune cell subset that detects viral nucleic acids and can induce off-target activity associated with the interferon (IFN) response through Toll-like receptors (TLRs).[25,26] Thus, the possibility of off-target activity needs to be excluded.

Macrophages play essential roles in the elimination of invading pathogens and clearance of dying cells.[28,29] They are known to be important for HIV infection. Macrophages and CD4$^+$ T cells have been reported as the major cellular reservoirs for latent HIV infection.[30–32] Unlike CD4$^+$ T cells, macrophages tend to contribute to long-term persistence of productive HIV infection because they are more resistant to the cytopathic effects of the virus and evade the defensive action of immune responses, with the progeny virus budding into and accumulating in their endocytic compartments designated as multivesicular bodies (MVBs).[30–32] The macrophage reservoir is established during the acute stages of HIV infection.[30,31,33] Macrophages also play an important role in the pathogenesis of HIV-1 infection. Because macrophages can cross the blood-tissue barrier, they are potent agents of HIV virus delivery to organs, including the brain.[30] They are particularly important in key immune-privileged sites, including the brain and the male reproductive tract.[34,35] The latter site is significantly enriched with infected macrophages compared with CD4$^+$ T cells and has the potential to be a major contributor to seminal fluid viral loads and/or cell-associated viruses that might permit HIV transmission when CD4$^+$ T cells are limiting.[36] Recent research has also revealed that HIV-infected macrophages can rapidly and efficiently transfer HIV to CD4$^+$ T cells.[37] Although most current studies are focusing on the CD4$^+$ T cell reservoir, functional cures will require targeting all of the substantial reservoirs, including macrophages. Therefore, HIV infection of macrophages plays an important role in viral pathogenesis and progression to AIDS.

Embryonic stem cells (ESCs) derived from a blastocyst have the capacity for self-renewal and for producing derivatives of all three germ layers.[38] They have enabled the study of early human development and *in vitro* analysis of differentiation. However, concerning the source for cellular therapy, issues of histocompatibility and ethical problems are associated with the use of human ESCs. Yamanaka and colleagues[39] showed that human somatic cells could be reprogrammed into pluripotency by simultaneous introduction of several factors to yield induced pluripotent stem cells (iPSCs). This technology enables avoiding issues associated with the use of human ESCs. Recent studies showed that human monocytic lineage cells were successfully derived from human iPSCs.[40,41] They have an advantage as an experimental model system of HIV infection over MDMs and monocytic cell lines, which have been typically utilized to study HIV infection. MDMs differentiated from peripheral blood monocyte cells (PBMCs) isolated from healthy donors[42] are highly variable owing to their inherent heterogeneity. Therefore, they lack in uniformity in the experimental protocol employed by different investigators. Conversely, monocytic cell lines such as THP-1 and U937 provide unlimited cell sources with genetic uniformity and ease of standardization. However, they do not reflect the HIV-1 infection model of macrophages *in vivo* as accurately as MDMs.[43] Macrophages derived from iPSCs provide an accurate experimental model system as MDMs and are also capable of unlimited and uniform expansion. In addition, MDMs have certain drawbacks, especially when using lentiviral vectors for gene therapy approaches. Obtaining high rates of lentiviral transduction in macrophages is difficult because of a range of host restriction factors such as SAMHD1.[44,45]

This study aimed to resolve the many current difficulties associated with the use of MDMs by using iPSC-derived macrophages as a relatively accessible source of essentially unlimited numbers of autologous differentiated macrophages. We transduced iPSCs with a lentiviral vector expressing an shRNA homologous to the nuclear factor κB (NF-κB)-binding region of HIV-1 promoter. These shRNA-treated iPSCs were successfully differentiated into macrophages. We then assessed the suppression of viral replication in these cells by using shRNA-mediated TGS with alterations in histone structure.

RESULTS

Generation of iPSCs Transduced with shRNA Targeting HIV Promoter

A previous study showed that shPromA, an shRNA homologous to the NF-κB-binding region of HIV-1 promoter, induced transcriptional suppression of HIV-1 in HIV-infected cells.[20,46,47] The vector backbone of shPromA is a self-inactivated lentiviral vector, which expresses an EGFP under the control of the Ubc promoter. To confirm that the transcriptional suppression induced was a consequence of sequence-specific suppression rather than that of off-target effects, we also designed shPromA-M2, which has two nucleotide mismatches within the shPromA target sequence (Figure 1A). To assess the inhibitory effects of shPromA in iPSC-derived

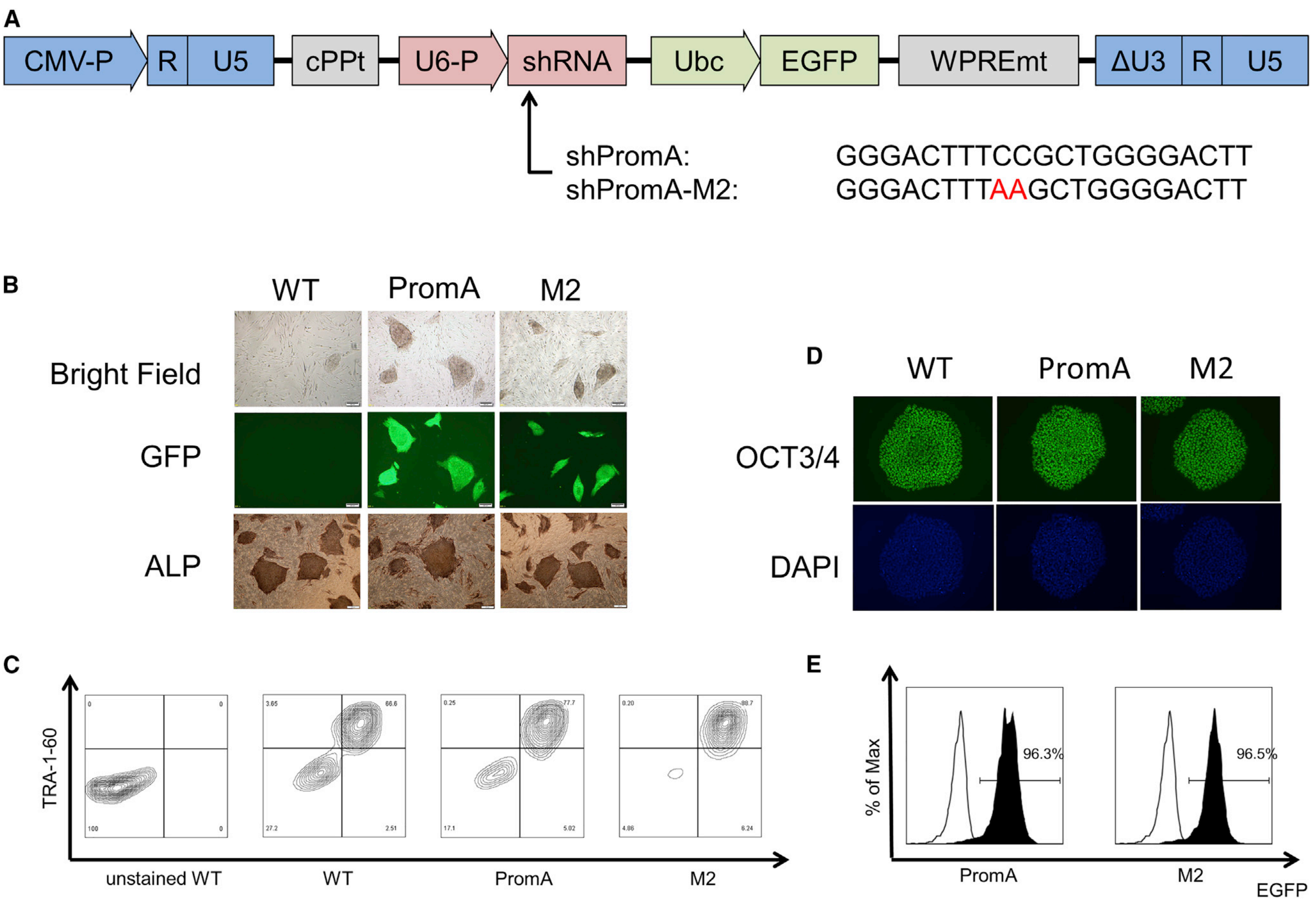

Figure 1. Generation of iPSCs Transduced with shRNA Targeting HIV Promoter

(A) Map of the short hairpin RNA (shRNA) in SIN lentivirus vector. SIN vector includes a central polypurine tract (cPPT), U6 promoter (U6 P), shRNA, ubiquitin C promoter (Ubc), and EGFP. WPREmt stands for mutant woodchuck promoter response element. Lack of the entire enhancer-promoter of the U3 region allows viral genome integration, but not expression. The sense, hairpin, and anti-sense sequence were inserted downstream of the U6 promoter sequence. Alignment of the shRNA targeting the NF-κB sites (shPromA) and the two-base mismatch control (shpromM2) is shown below. The red text in the alignment of shPromM2 highlights nucleic acids that differ from the shPromA sequence. (B) The iPSC lines were observed by alkaline phosphatase (ALP) staining or fluorescence microscopy after transduction with shPromA (PromA), shPromA-M2 (M2), and control (wild-type [WT]). Scale bar, 200 μm. (C) Flow cytometric analysis of human SSEA-4 and TRA-1-60 on unstained untransduced iPSCs, untransduced iPSCs, and iPSCs transduced with shPromA and with shPromA-M2 (unstained WT, WT, PromA, and M2, respectively). (D) iPSC lines were fixed by ethanol, stained by anti-OCT3/4 antibodies, and analyzed by fluorescence microscopy for OCT3/4 expression after transduction with shPromA (PromA), shPromA-M2 (M2), and control (WT). (E) iPSCs transduced with shPromA (PromA) and shPromA-M2 (M2) were analyzed for EGFP expression by using flow cytometer (black). Untransduced iPSCs are shown as negative controls (white).

macrophages, we used an iPSC line established by reprogramming of T cell as described before[48] in this study. We transduced iPSCs with shPromA and shPromA-M2. The morphologies of the original iPSCs and those transduced with shPromA and shPromA-M2 are shown in Figure 1B. EGFP expression was confirmed by fluorescence microscopy (Figure 1B). We also confirmed the sustained expression of alkaline phosphatase (Figure 1B). All iPSC lines were positive for SSEA-4, TRA-1-60, and OCT3/4 sufficient markers of pluripotency[49] (Figures 1C and 1D). We then evaluated the transduction efficiency of these vectors in iPSCs. At an MOI of 1, transduction efficiency of 96.3% for shPromA and 96.5% for shPromA-M2 was achieved as determined by FACS analysis for EGFP expression at 8 days after transduction (Figure 1E). Only one of the chromosomal abnormalities (47XXX) occurring in pluripotent stem cells was identified during *in vitro* culture in whole shPromA and shPromA-M2 transduction experiments (Figure S1A). By simple sub-cloning technique to select good-shaped colonies, we were able to eliminate abnormal karyotype iPSCs. We confirmed that frequency of presence of abnormal karyotype iPSCs by X chromosome fluorescence *in situ* hybridization (FISH) assay after sub-cloning was 0.7% in 1,000 cells, which was similar to levels in PBMCs obtained from healthy donor (Figure S1B). These observations confirmed the pluripotency of the iPSCs expressing shPromA and shM2. The shPromA and shPromA-M2 harboring lentiviral vector in iPSCs and iPSC-derived macrophages were identified using genomic PCR analysis (Figure S2).[47]

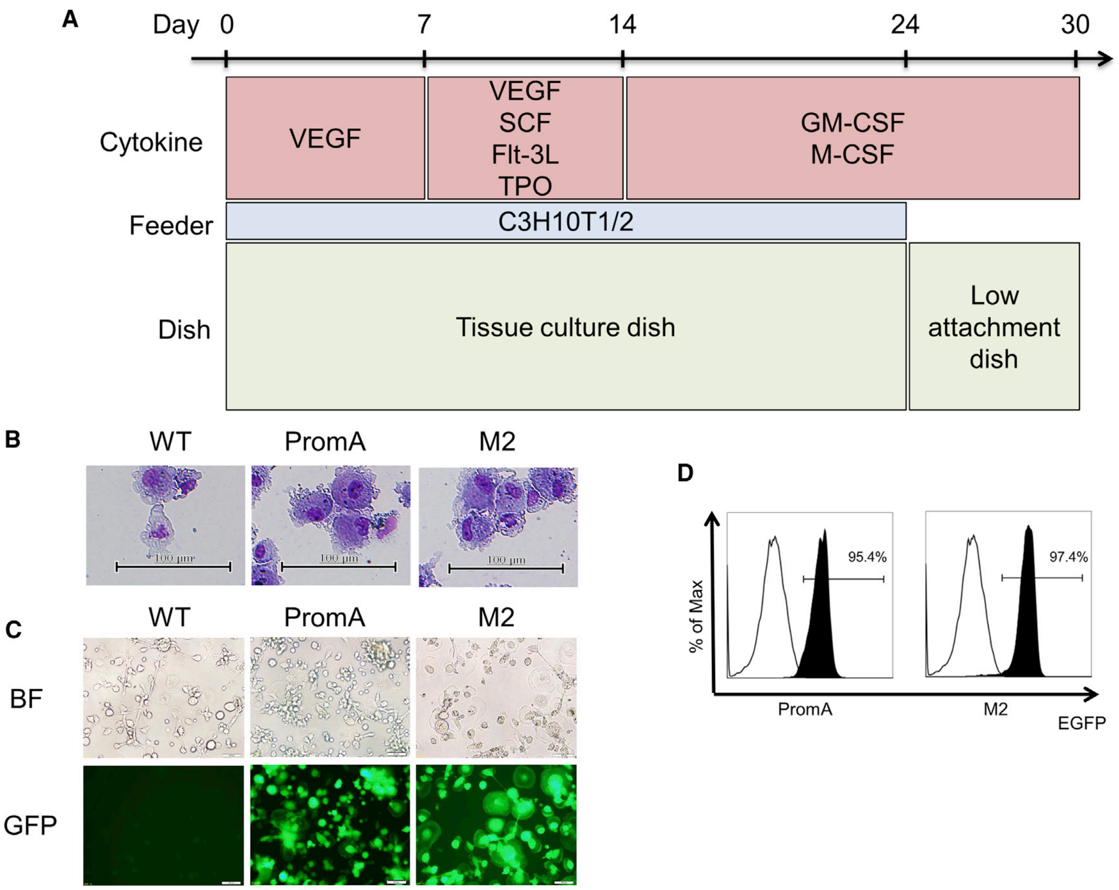

Figure 2. Differentiation of Human iPSCs into HSCs and Macrophages *In Vitro*

(A) *In vitro* macrophage differentiation protocol. (B) May-Giemsa staining of macrophages derived from shPromA (PromA), shPromA-M2 (M2), or original iPSCs (wild-type [WT]) with an image of monocyte-derived macrophage (MDM). (C) iPSCs transduced with shPromA (PromA), shPromA-M2 (M2), and original iPSCs (WT) were differentiated into macrophages. EGFP expression was analyzed using fluorescence microscopy. (D) Macrophages derived from iPSCs transduced with shPromA (PromA) and shPromA-M2 (M2) were analyzed for EGFP expression by using flow cytometer (black). Macrophages derived from wild-type iPSCs are shown as negative controls (white).

Differentiation of Human iPSCs into $CD34^+$ Hematopoietic Progenitors and Macrophages *In Vitro*

Following the application of specific *in vitro* differentiation protocols, iPSCs are differentiated into macrophages.[40,41] This protocol was applied to assess the capacity of iPSCs expressing shPromA and shPromA-M2 for macrophage differentiation. For the generation of hematopoietic stem and/or progenitor cells, iPSCs were co-cultured on C3H10T1/2 feeder cells in the presence of vascular endothelial growth factor (VEGF) under 5% oxygen until day 7 and then in the presence of VEGF, FLT-3L, stem cell factor (SCF), and TPO under 20% oxygen until day 14.[48] In this step, we modified the protocol slightly by supplementing TPO, which has been indicated to play an important role in the expansion of $CD34^+$ cells,[46,50] and optimized its concentration (data not shown). Dome-like colonies appeared by days 3–4. Cystic structures were observed after day 10. For the generation of macrophages, adherent cells were removed, and non-adherent cells were co-cultured on C3H10T1/2 feeder cells in the presence of granulocyte-macrophage colony-stimulating factor (GM-CSF) and macrophage colony stimulating factor (M-CSF) on day 14 of culture. On day 24, adherent cells were transferred and cultured in the presence of GM-CSF and M-CSF without C3H10T1/2 feeder cells. On day 30, morphologically distinct macrophages were observed to be attached to the culture plate. This protocol is shown in Figure 2A.

Characterization of Macrophages Generated from iPSCs

Macrophages derived from iPSCs expressing shPromA and shPromA-M2 showed adherent, polygonal, and spindle shape under a microscope (Figures 2B and 2C). Transgene expression efficiency in iPSC-derived macrophages was determined by EGFP expression. It was extremely high in iPSC-derived macrophages (95.4% with shPromA, 97.4% with shPromA-M2) compared with EGFP expression levels achieved using MDMs (average transduction level 20%, due to the host restriction factors within MDMs).[51] The iPS-derived macrophages expressed macrophage-associated markers such as CD11b, CD11c, CD86, and HLA-DR and retained the expression

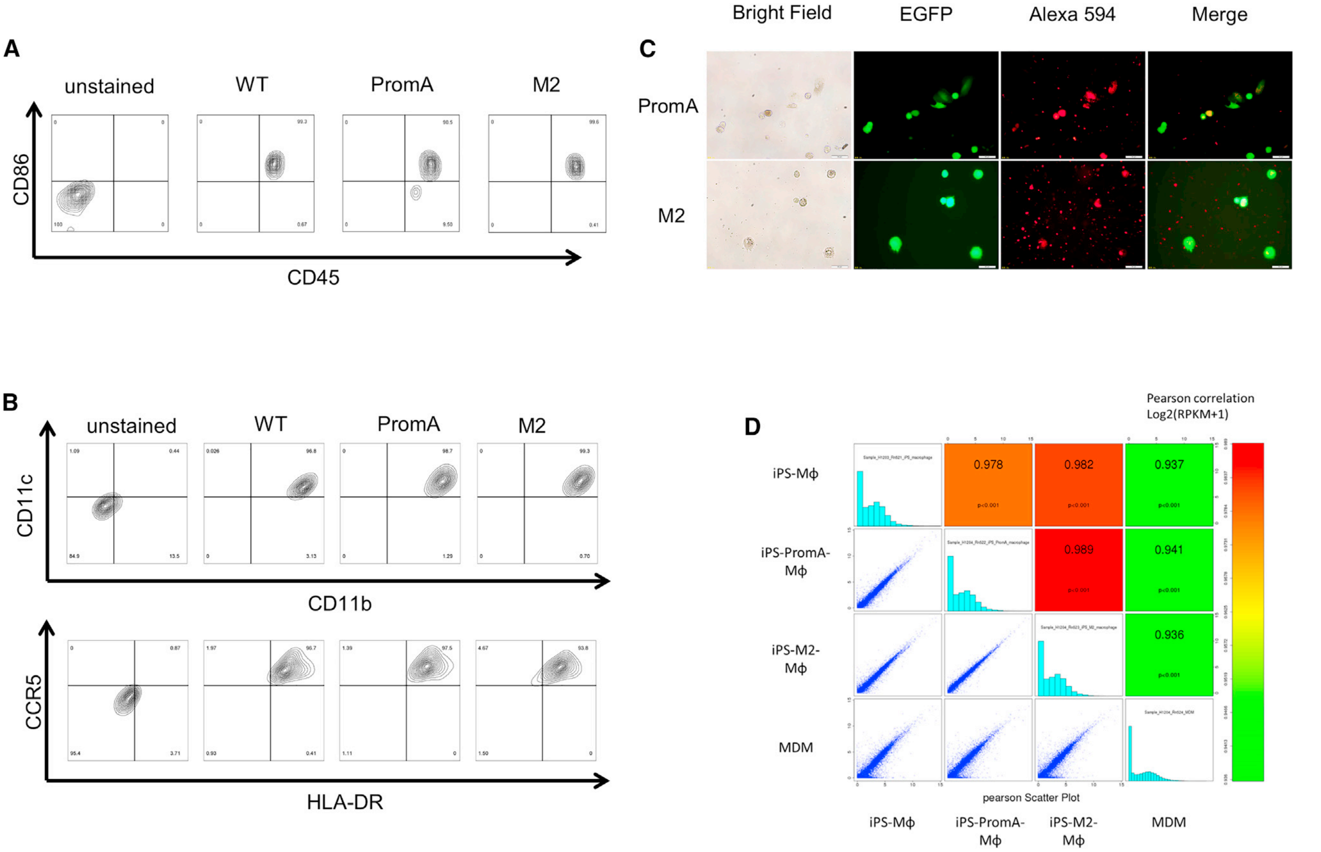

Figure 3. Characterization of Macrophages Generated from iPSCs

(A) Flow cytometric analysis of human CD45, CD86 on macrophages derived from peripheral monocytes, control iPSCs, and iPSCs transduced with shPromA or shPromM2 (MDM, WT, PromA, and M2, respectively). (B) Flow cytometric analysis of human CD11b, CD11c, HLA-DR, and CCR5 on macrophages derived from iPSCs transduced with shPromA or shPromM2, and control iPSCs (PromA, M2, and WT, respectively). (C) Macrophages (green) derived from iPSCs transduced with shPromA (upper) and shPromA-M2 (lower) were incubated with Alexa Flour 594 *Escherichia coli* (red). The cells were microscopically observed after incubation for 1 hr. BioParticles were co-localized in macrophages (yellow in merged pictures). Scale bar, 50 μm. (D) Global gene expression of macrophages derived from iPSCs or MDM was analyzed. Heatmaps show the correlation coefficients between samples.

level of CD4 and CCR5 that act as receptors for HIV-1 entry (Figures 3A and 3B; Figure S3). Phagocytic function of iPSC-derived macrophages was confirmed using Alexa Flour 594-conjugated *Escherichia coli* BioParticles. As observed in the fluorescence microscopic image shown in Figure 3C, we detected co-localization of captured BioParticles in iPS-derived macrophages after 1 hr of co-culture.

In addition, we investigated whether the global gene expression profile of iPSC-derived macrophages and MDMs showed any differences. Transcriptome analysis by mRNA sequencing indicated that iPSC-derived and TGS-modified macrophages retained similar mRNA expression profile to that of MDMs (Figure 3D).

Taken together, these findings suggest that macrophages derived from iPSCs were comparable with primary macrophages in morphology, cell surface markers, and global gene expression profiles, which indicated that these cultures could provide susceptible target cells for HIV infection.

Suppression of Viral Replication in iPS-Derived Macrophages by Using shPromA

Next, we assessed whether iPSC-derived macrophages by using shPromA would be resistant to CCR5-tropic HIV infection. To evaluate protection from HIV infection, we challenged iPSC-derived macrophages with HIV-1 Ba-L virus, which uses CCR5 as well as CD4 for viral entry. We extracted genomic DNA from infected iPSC-derived macrophages after day 4 in three experimental groups and confirmed the presence of HIV-1 DNA across all three experimental groups by using PCR (Figure 4A). We then measured transcriptional activity to compare the HIV-1 transcription level from the HIV-1 promoter by measuring HIV RNA from the infected iPSC-derived macrophages at days 4 and 7 after HIV infection (Figure 4B). The data clearly showed that iPSC-derived macrophages transfected with shPromA inhibit HIV-1 transcription with around 10 and 20 times reduction compared with that in iPSC-derived macrophages transfected with shPromA-M2 and the mock control, respectively, at day 11. To confirm this reduction, we also measured

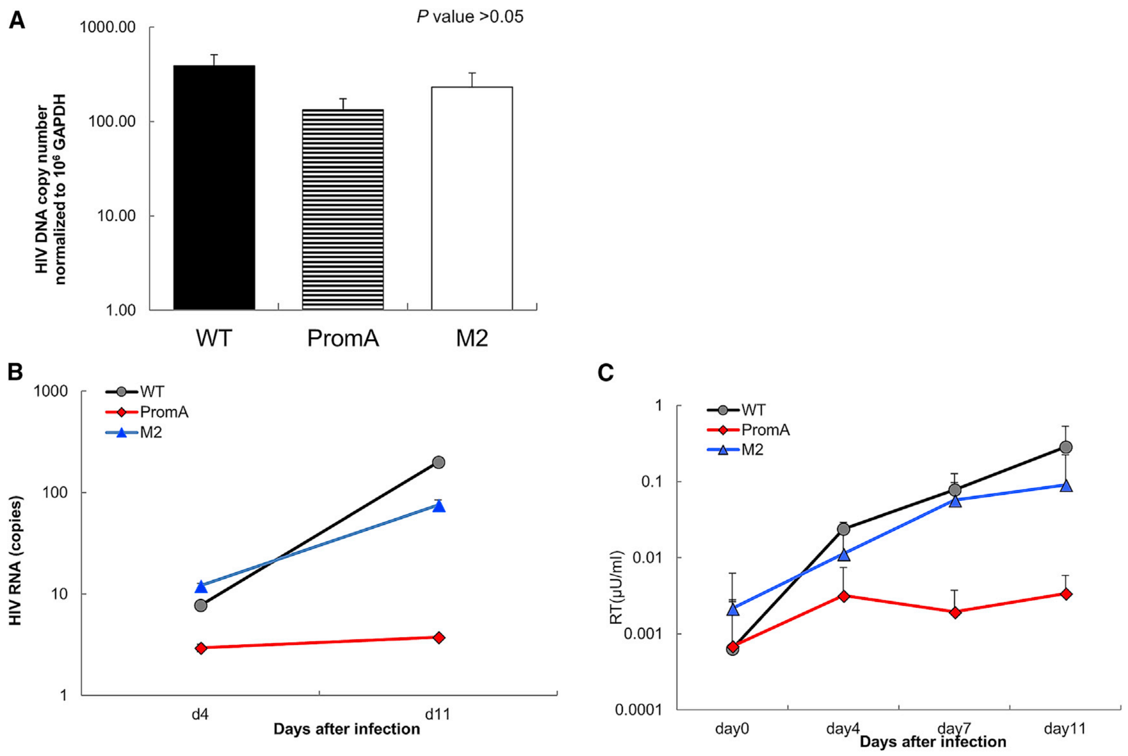

Figure 4. Suppression of Viral Replication in iPS-Derived Macrophages Expressing shPromA

Macrophages derived from iPSCs transduced with shPromA or shPromM2, and control iPSCs (PromA, M2, and WT, respectively) were challenged with an R5-tropic HIV-1 Ba-L virus. (A) Real-time PCR of the gag region confirms similar levels of integrated HIV-1 provirus presented in all samples. However, HIV-1 replication was inhibited in PromA, as determined by (B) viral mRNA levels normalized to GAPDH and (C) viral reverse transcriptase activity. Two-way ANOVA revealed statistically significant interaction between transduction of shRNA and viral mRNA levels or reverse transcriptase activity ($p < 0.01$).

the amount of virus released from HIV-1-infected iPSC-derived macrophages in culture supernatants by measuring the reverse transcriptase (RT) activity.[52] At day 4 post-transfection, shPromA-transduced iPSC-derived macrophages showed reduction in virus production compared with those in control cultures (Figure 4C). This reduction was sustained at least until day 11. Taken together, these results indicate the inhibitory effect of shPromA on HIV-1 infection in iPSC-derived macrophages, which is consistent with our previous experimental data in MDMs.[46,47,53]

shPromA Suppressed Viral Replication through TGS

Several studies have shown that TGS mediated by siRNA targeted to the promoter region is associated with the changes in histone methylation and deacetylation status around the NF-κB-binding region.[20,53,54] Therefore, we investigated the alteration in histone methylation status in our model. We performed chromatin immunoprecipitation (ChIP) assays by using antibodies to histone 3 lysine 27 trimethylation (H3K27me3) on day 8 post-HIV infection. Macrophages transduced with shPromA exhibited a significant increase in H3K27me3 compared with those in the controls (Figure 5A). In addition, we assessed the histone 3 lysine 9 acetylation (H3K9Ac) and confirmed that treatment with shPromA was followed by failure of H3K9Ac enrichment compared with that in controls (Figure 5B). These data indicated that shPromA-induced TGS of HIV is associated with histone methylation and histone deacetylation, particularly induction of H3K27me3 and reduction of H3K9Ac. These data are also consistent with our previous *in vitro* experimental data based on shPromA lentivirus-transduced cell lines.[19,47,53,55]

No Significant Change Is Observed in the Expression of NF-κB-Driven Genes by shPromA

Previous studies revealed that long single-strand and double-strand RNAs are detected by immune cells specialized to recognize viral nucleic acids through TLR3, TLR7, and TLR8, leading to the triggering of the IFN pathway and induction of non-specific off-target effects.[25,26] shPromA is a long single-strand RNA homologous to the NF-κB-binding region in HIV long terminal repeat (LTR); it might potentially alter the expression of other NF-κB-driven genes and induce sequence-specific off-target effects.[27] RNA sequencing of shPromA-transduced iPSC-derived macrophages was used to exclude the sequence-specific or non-specific off-target effects mediated by shPromA. We examined the transcription level of 86 NF-κB-driven genes, including IFN genes (Table S1), but did not detect any significant difference in the transcription of these NF-κB-driven genes (Figures 6A–6C). The data were concordant with a previous report that shPromA does not alter the expression

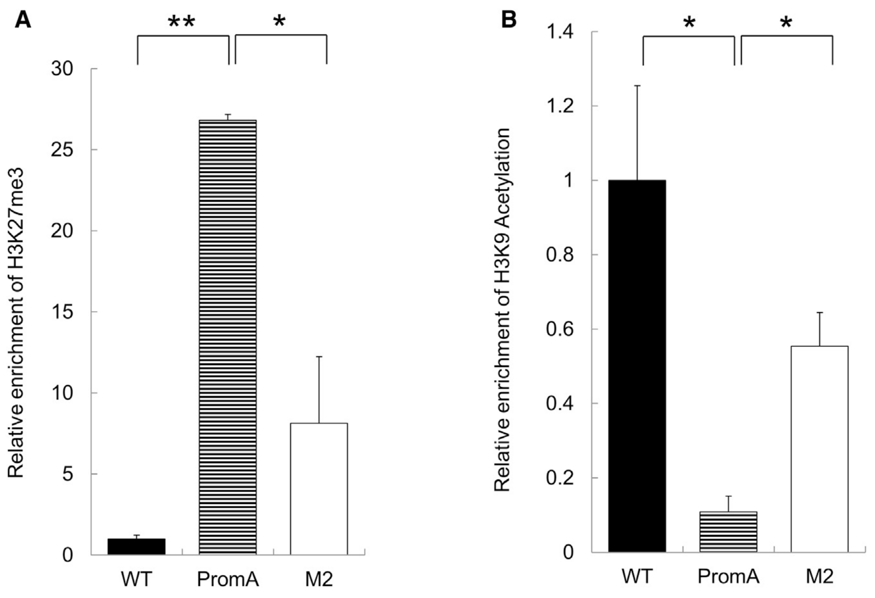

Figure 5. shPromA Suppressed Viral Replication through TGS

Chromatin immunoprecipitation assays were performed on macrophages derived from iPSCs transduced with shPromA or shPromM2, and control iPSCs (PromA, M2, and WT, respectively) at day 4. DNA fragments from whole-cell extracts were co-precipitated against (A) histone 3 lysine 9 acetylation (H3K9Ac) and (B) histone 3 lysine 27 trimethylation (H3K27me3). Each value shown is the relative enrichment normalized to the value obtained from mock transfection. $^{*}p < 0.05$; $^{**}p < 0.01$.

of other NF-κB-driven genes by targeting other NF-κB-binding motifs in host genes.[47] These results strongly support that the observed HIV-1 suppression is not a result of off-target effects induced by shPromA.

DISCUSSION

Previous studies have shown TGS of HIV-1 in HeLa or T cell lines by using shRNA targeting the HIV-1 promoter region through the induction of epigenetic modifications.[56,57] Suppression of viral replication through the TGS pathway was also observed in an *in vivo* NOD/Scid/Jak3-deficient (NOJ) humanized mouse model.[47] In this study, we showed *HIV-1* gene silencing via the TGS pathway in iPSC-derived macrophages. The target sequence of shRNA mediating TGS was located within the NF-κB-binding region of the HIV-1 promoter, which induces the activation of the HIV-1 LTR response to NF-κB inducers.[58] Expression of the promoter-targeted shRNA did not show any adverse effects on the morphology of iPSCs, which showed round colonies and tight edges, as shown in Figure 1B. Moreover, iPSCs expressing promoter-targeted shRNA were positive for markers of pluripotency as measured by immunofluorescence. Therefore, the expression of promoter-targeted shRNA did not affect their pluripotency or morphology.

Another major concern with transduction of shRNA-expressing vectors is the possibility of their effect on differentiation into phenotypically and functionally normal macrophages. Following the application of specific *in vitro* differentiation protocols, shRNA-expressing iPSCs were successfully differentiated into macrophages at a rate similar to that of control iPSCs. The macrophages derived from iPSCs were comparable with MDMs with respect to morphology, cell surface markers, and phagocytic capability. We observed no gene silencing of EGFP throughout their differentiation into macrophages.

Upon challenge with HIV-1 infection in iPSC-derived macrophages expressing shPromA compared with that in original iPSC-derived macrophages and iPSC-derived macrophages with two-base mismatched control (shPromA-M2), we observed strong suppression of HIV-1 transcriptional activity measured using viral mRNA analysis. We also found strong reduction in viral replication measured using RT assay by using culture supernatants. We confirmed that this inhibitory effect on viral replication was due to the induction of TGS with alteration in histone methylation and acetylation status. Some studies have shown sequence-specific or sequence-nonspecific off-target effects of siRNAs designed to induce TGS.[25–27] We confirmed that the expression of promoter-targeted shRNA did not have any significant off-target effects, as determined by the expression analysis of 86 NF-κB-driven genes, including IFN genes.

Viral entry of HIV-1 requires binding to a CD4 receptor and either CCR5 or CXCR4. Homozygosis for a 32-bp deletion in the CCR5 allele confers high resistance against HIV-1 acquisition,[59] which makes CCR5 an attractive target for gene therapy of HIV. A previous study showed allogeneic stem cell transplantation in a patient with HIV infection and acute myeloid leukemia from a donor who was homozygous for CCR5 delta32. In this patient, no active, replicating HIV could be detected for 20 months after transplantation and discontinuation of antiretroviral therapy.[60] This result indicates that the generation of immune cells resistant to HIV infection can be a potential therapeutic application for the treatment of chronic HIV infections.

For generating HIV-resistant immune cells, previous studies have used CCR5 gene modification in HSPCs, $CD4^{+}$ T lymphocytes, and iPSCs and showed their resistance against viral infection.[61–63] However, CCR gene modification in immune cells cannot prevent the advent of non-CCR5-tropic variants, as observed in a patient at 20 months after allogeneic stem cell transplantation of CCR5 delta32.[60] In addition, CCR3, as well as CCR5, has been reported to promote efficient infection of the CNS by HIV-1.[64] These data strongly suggest that gene therapy approaches can inhibit HIV-1 entry into human cells. However, less impact would be observed on the

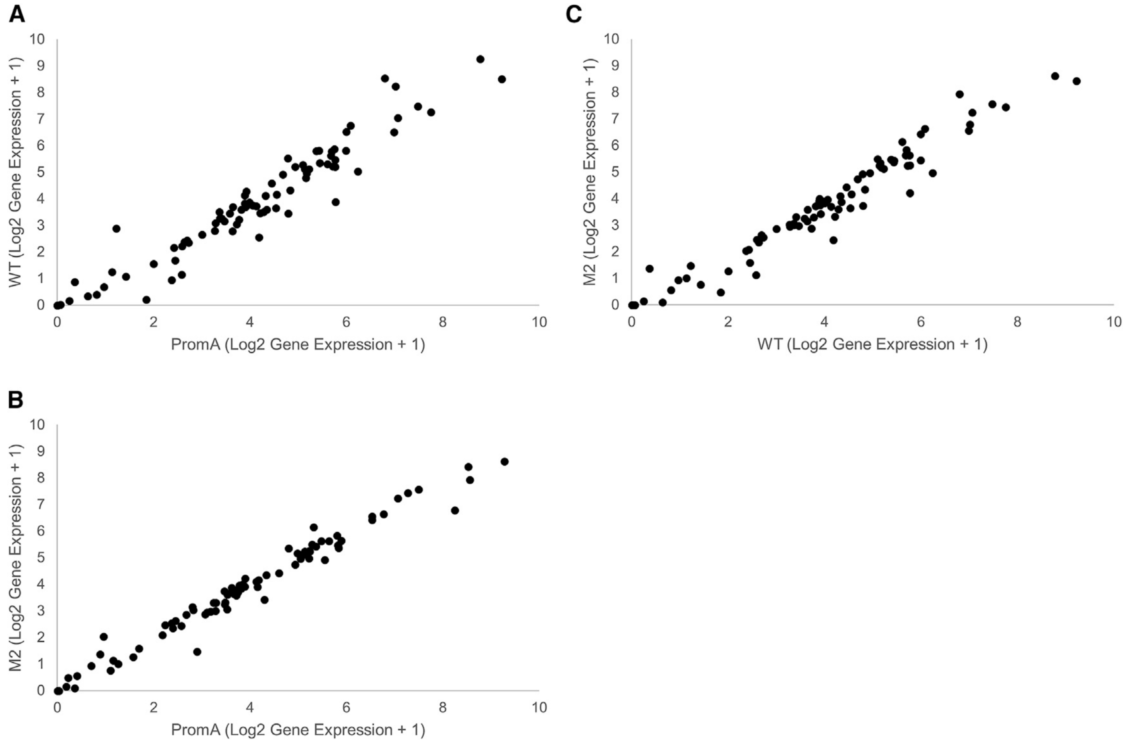

Figure 6. RNA Sequence of the 86 NF-κB-Driven Genes, Including the IFN Genes

Scatterplot of log transformation of the relative expression of mRNA from 86 NF-κB-driven genes. No significant difference was noted in the relative expression (A) between macrophages derived from untransduced iPSCs (wild-type [WT]) and those derived from iPSCs transduced with shPromA (PromA); (B) between macrophages derived from iPSCs transduced with shPromA (PromA) and those derived from iPSCs transduced with shPromA-M2 (M2); and (C) between macrophages derived from iPSCs transduced with shPromA-M2 (M2) and those derived from untransduced iPSCs (WT).

reduction of the established latently infected reservoir population when these approaches targeting HIV-1 entry inhibition are used. Therefore, our shRNA-mediated TGS approach is an alternative potent candidate gene therapy for HIV-1 infection aiming to control HIV-1 transcription under a silenced mode as observed in latent HIV-1 infection.

The iPSC-derived macrophage model was utilized to confirm the inhibitory effect of shPromA delivered by a lentiviral vector. To further elucidate this approach, we also intend to investigate the use of T cells derived from iPSCs at the double-positive (DP) stage. Previously, we established a culture protocol to generate T cells at the DP stage.[48] Following the application of our protocol, shPromA-treated iPSCs will be differentiated into T cells at the DP stage. If viral suppression is recapitulated in iPSC-derived T cells at the DP stage, an increased emphasis on aiming to achieve functional care by iPSC-based gene therapy will be strongly suggested.

We also observed that transduction efficiency in iPSC-derived macrophages after transfection with the lentiviral vector, as determined by GFP-expressing cells, was extremely high compared with the GFP expression levels obtained when MDMs were used. We usually achieve transfection efficiency of around 20% because of host restriction factors such as SAMHD1. We also expect a high level of transduction efficiency in differentiated T cells at the DP stage derived from shPromA-transfected iPSCs.

In this study, we showed the inhibitory effect of promoter-targeted shRNA on HIV-1 replication in iPSC-derived macrophages through the TGS pathway. This concept is not limited to macrophages derived from iPSCs and might be extended to T cells at the DP stage or even to hematopoietic stem cells (HSCs) derived from iPSCs, if optimization of differential conditions can be achieved. Generation of HSCs derived from shPromA-treated iPSCs might enable the reconstitution of the immune system with immune cells resistant to HIV-1 infection. The data presented in this study emphasize the advantages of generating HIV-1-resistant HSCs derived from iPSCs as therapeutic cells for HIV gene therapy.

MATERIALS AND METHODS

Human iPSCs

In this study, we used the human iPSC line 4GAD1-4 obtained from a monoclonal T cell clone specific to glutamic acid decarboxylase (GAD) by using a Sendai virus vector, as described previously.[48] iPSCs were maintained on tissue culture dishes coated with mouse embryonic fibroblasts (MEFs) or Matrigel (Becton Dickinson) in

mTeSR1 serum-free medium (STEMCELL Technologies). Some PBMC samples were obtained from healthy volunteers who provided written informed consent. Research was approved by the Kyoto University School of Medicine ethical committee (no. G590).

Construction of shRNA-Expressing Lentiviral Vector

The construction of lentiviral vectors by using the following sense and antisense DNA oligomers has been described previously[47]: shPromA sense, 5′-GAT CCG GGA CTT TCC GCT GGG GAC TTC TGT GAA GCC ACA GAT GGG AAG TCC CCA GCG GAA AGT CCC TTT TTT AT-3′; shPromA antisense, 5′-CGA TAA AAA AGG GAC TTT CCG CTG GGG ACT TCC CAT CTG TGG CTT CAC AGA AGT CCC CAG CGG AAA GTC CCG-3′ and shM2 sense, 5′-GAT CCG GGA CTT TAA GCT GGG GAC TTC TGT GAA GCC ACA GAT GGG AAG TCC CCA GCG GAA AGT CCC TTT TTT AT-3′; shM2 antisense, 5′- CGA TAA AAA AGG GAC TTT AAG CTG GGG ACT TCC CAT CTG TGG CTT CAC AGA AGT CCC CAG CTT AAA GTC CCG-3′.

Lentiviral Transduction into iPSCs

iPSCs at 5×10^5 cells on Matrigel-coated culture plates were suspended in 100 μL of StemFit (Ajinomoto). After lentiviral suspension (MOI = 1) was added, these tubes were rotated at 37°C for 20 min by using a Macsmix Tube Rotator (Miltenyi Biotec). After rotation, the iPSCs were transferred to Matrigel-coated culture plates in StemFit culture medium supplemented with Y-27632 Rho kinase inhibitor (ROCKi; Tocris, Bristol, UK). After a few days of culture, GFP-positive iPSCs were sorted by flow cytometry analysis.

Hematopoietic Differentiation and Generation of Macrophages from iPSCs

To induce hematopoietic differentiation from human iPSCs, we slightly modified a previously described method.[65] In brief, small clumps (<100 cells) of iPSCs maintained on MEFs were collected and co-cultured on C3H10T1/2 cells in embryoid body (EB) medium (Iscove's modified Dulbecco's medium) containing VEGF (20 ng/mL). On day 7, SCF (50 ng/mL), FLT-3L (50 ng/mL), and/or TPO (2 or 20 ng/mL) was added to the culture. On day 14, hematopoietic progenitor cells in the iPSC sacs were collected and transferred to a newly prepared C3H10T1/2 layer in the presence of M-CSF (50 ng/mL) and GM-CSF (50 ng/mL). On day 24, after the floating and loosely adherent cells were removed, firmly adherent cells were collected and transferred to low-attachment six-well culture plates (Corning Costar Ultra-Low attachment multiwell culture plates; Sigma-Aldrich) in the presence of GM-CSF (50 ng/mL) and M-CSF (50 ng/mL), and they showed macrophage-like morphology with fringes within 5 days.

Flow Cytometry

Flow cytometric data were acquired using FACScan (BD Biosciences, Bedford, MA, USA) and then analyzed using FlowJo software (Tree Star). The following monoclonal antibodies (mAbs) were purchased from BD Pharmingen (San Diego, CA, USA), Beckman Coulter (Miami, FL, USA), eBioscience (San Diego, CA, USA), Miltenyi Biotec (Auburn, CA, USA), R&D Systems (Minneapolis, MN, USA), or Caltag Laboratories (Hamburg, Germany): SSEA4-PE, TRA-1-60-Alexa Flour 648, CD45-allophycocyanin (APC)-Cy7, CD14-PB, CD11b-PE, CD11c-APC, CD68-APC, HLA-DR-BV605, and CCR-PerCP-Cy5.5.

Analysis of Phagocytosis Function in Myeloid Lineage Cells Derived from iPSCs

Macrophages derived from iPSCs were incubated for 1 hr with BioParticles (*E. coli* strain K-12 BioParticles, Alexa Flour 594 conjugated, catalog [Cat] #E-23370; Life Technologies), washed three times with PBS, and observed using an IX71 inverted microscope (Olympus).

HIV-1 Infection and Viral Quantification

iPSC-derived macrophages (1×10^5) were infected with HIV-1 Ba-L virus (1 ng p24 virus), and infection was allowed to establish for 2 hr.[55] qPCR was performed to analyze the HIV-1 DNA levels. Infected cells were collected 24 hr after infection, and genomic DNA was extracted (QIAamp DNA Blood Mini Kit; QIAGEN). The integrated HIV-1 genome was analyzed using real-time PCR by using a TaqMan probe with a standard curve generated using serial dilutions of the HIV-1 molecular clone plasmid (pNL4-3, Cat No.114; NIH AIDS Reagent Program). The following primers were used: HIV gag forward primer, 5′-AGTGGGGGGACATCAAGCAGCCATG CAAAT-3′; HIV gag reverse primer, 5′-TACTAGTAGTTCCTGC TATGTCACTTCC-3′. On days 0, 4, 7, and 11, tissue culture supernatants were sampled and analyzed for reverse transcriptase activity, as described previously.[52] The levels of HIV-1 mRNA were measured using qRT-PCR by using the HIV gag primers (shown above). Levels of GAPDH mRNA were measured as an internal control. To plot standard curves, we infected PM-1 cells (8×10^6 cells) with HIV-1 (NL4-3, 100 ng p24).

ChIP Assay

On day 4 after infection, macrophages were collected, and a ChIP assay was performed using a ChIP assay kit (Merck Millipore), as described previously.[53] In brief, cultures were fixed with 1% formaldehyde for 10 min at room temperature and incubated for 5 min with 0.125 mol/L glycine; after three washes with cold PBS, the pellets were resuspended in 400 μL of SDS with protease inhibitor cocktail (Roche). The lysate was then sonicated to shear DNA for 4 min by using a 90-W ultrasonic processor (Qsonica Sonicator Q700; Qsonica, Farmingdale, NY, USA). The lysate was then centrifuged at $10{,}000 \times g$ for 5 min. The resulting supernatant was divided into three aliquots, and anti-acetylation-H3K9 (Millipore), anti-trimethylation H3K27 (Millipore), and no antibody (control) were added to the respective aliquots. Samples were incubated with Protein A-Agarose beads (Upstate, Charlottesville, VA, USA). The beads were then washed, and DNA was extracted using the QIAprep PCR extraction kit (QIAGEN). Real-time PCR analysis was performed using a SensiFAST SyBr kit (Bioline, NSW, Australia). The following primers were used: LTR forward, 5′-TACAAGGGACTTTCCGCTGG-3′; LTR reverse, 5′-TTGAGGCTTAAGCAGTGGG-3′. The PCR conditions were as follows: 94°C for 2 min, followed by 45 cycles of 95°C for

7 s and 58°C for 20 s. To generate a standard curve, we used diluted aliquots of HIV-1 molecular clone plasmid (pNL4-3). The results of the PCR in aliquots with no antibody were subtracted from the results of interest.

RNA Sequencing

mRNA from the macrophages was extracted using an RNeasy mini kit (QIAGEN). cDNA was synthesized using SMARTer Ultra Low Input RNA and sequenced using an Illumina Sequencing-HV kit (Clontech, Mountain View, CA, USA), after which the Illumina library was prepared using a Low Input Library Prep kit (Clontech). The libraries were sequenced using HiSeq 2500 in 101 cycle Single-Read mode. All sequence reads were extracted in FASTQ format by using BCL2FASTQ Conversion Software 1.8.4 in the CASAVA 1.8.2 pipeline. The sequence reads were mapped to hg19 reference genome, downloaded on December 10, 2012, by using TopHat v2.0.8b,[66] and quantified using RPKMforGenes.[67]

SUPPLEMENTAL INFORMATION

Supplemental Information includes three figures and one table and can be found with this article online at https://doi.org/10.1016/j.omtn.2018.07.017.

AUTHOR CONTRIBUTIONS

K.S. and S. Kaneko conceived and designed the experiments. K.H., M.H., A.K.-T., and A.W. performed the experiments or analyzed the data. K.H., K.S., and S. Kaneko drafted and edited the manuscript. S.I., N.U., W.B., and S. Kamibayashi provided technical support. H.N. provided critical materials, including iPSCs. All authors reviewed the manuscript.

CONFLICTS OF INTEREST

S. Kaneko received research funding from Kyowa Hakko Kirin Co. Ltd., Takeda Pharmaceutical Co. Ltd., Sumitomo Chemical Co. Ltd., and Thyas Co. Ltd.

ACKNOWLEDGMENTS

The authors thank Dr. Yasushi Uemura (National Cancer Center, Japan) for giving advice of myeloid cell differentiation from pluripotent stem cells. This study was partially supported by grants from Core Center for iPS Cell Research of Research Center Network for Realization of Regenerative Medicine by the Japan Agency for Medical Research and Development (S. Kaneko), Grants-in-Aid 25114707 “Carcinogenic Spiral” by the Japanese Ministry of Education, Culture, Sports, Science, and Technology (S. Kaneko), and St Vincent’s Clinical Grant (K.S.). The study was performed at the Center for iPS Cell Research and Application (CiRA), Kyoto University, Sakyo-ku, Kyoto, Japan.

REFERENCES

1. Palella, F.J., Jr., Delaney, K.M., Moorman, A.C., Loveless, M.O., Fuhrer, J., Satten, G.A., Aschman, D.J., and Holmberg, S.D.; HIV Outpatient Study Investigators (1998). Declining morbidity and mortality among patients with advanced human immunodeficiency virus infection. N. Engl. J. Med. *338*, 853–860.
2. Finzi, D., and Siliciano, R.F. (1998). Viral dynamics in HIV-1 infection. Cell *93*, 665–671.
3. Han, Y., Wind-Rotolo, M., Yang, H.C., Siliciano, J.D., and Siliciano, R.F. (2007). Experimental approaches to the study of HIV-1 latency. Nat. Rev. Microbiol. *5*, 95–106.
4. Finzi, D., Hermankova, M., Pierson, T., Carruth, L.M., Buck, C., Chaisson, R.E., Quinn, T.C., Chadwick, K., Margolick, J., Brookmeyer, R., et al. (1997). Identification of a reservoir for HIV-1 in patients on highly active antiretroviral therapy. Science *278*, 1295–1300.
5. Esté, J.A., and Cihlar, T. (2010). Current status and challenges of antiretroviral research and therapy. Antiviral Res. *85*, 25–33.
6. Carr, A. (2003). Toxicity of antiretroviral therapy and implications for drug development. Nat. Rev. Drug Discov. *2*, 624–634.
7. Martinez-Picado, J., DePasquale, M.P., Kartsonis, N., Hanna, G.J., Wong, J., Finzi, D., Rosenberg, E., Gunthard, H.F., Sutton, L., Savara, A., et al. (2000). Antiretroviral resistance during successful therapy of HIV type 1 infection. Proc. Natl. Acad. Sci. USA *97*, 10948–10953.
8. Capodici, J., Karikó, K., and Weissman, D. (2002). Inhibition of HIV-1 infection by small interfering RNA-mediated RNA interference. J. Immunol. *169*, 5196–5201.
9. Coburn, G.A., and Cullen, B.R. (2002). Potent and specific inhibition of human immunodeficiency virus type 1 replication by RNA interference. J. Virol. *76*, 9225–9231.
10. Jacque, J.-M., Triques, K., and Stevenson, M. (2002). Modulation of HIV-1 replication by RNA interference. Nature *418*, 435–438.
11. Lee, N.S., Dohjima, T., Bauer, G., Li, H., Li, M.J., Ehsani, A., Salvaterra, P., and Rossi, J. (2002). Expression of small interfering RNAs targeted against HIV-1 rev transcripts in human cells. Nat. Biotechnol. *20*, 500–505.
12. Stevenson, M. (2003). Dissecting HIV-1 through RNA interference. Nat. Rev. Immunol. *3*, 851–858.
13. Boden, D., Pusch, O., Lee, F., Tucker, L., and Ramratnam, B. (2003). Human immunodeficiency virus type 1 escape from RNA interference. J. Virol. *77*, 11531–11535.
14. Das, A.T., Brummelkamp, T.R., Westerhout, E.M., Vink, M., Madiredjo, M., Bernards, R., and Berkhout, B. (2004). Human immunodeficiency virus type 1 escapes from RNA interference-mediated inhibition. J. Virol. *78*, 2601–2605.
15. Wassenegger, M., Heimes, S., Riedel, L., and Sänger, H.L. (1994). RNA-directed de novo methylation of genomic sequences in plants. Cell *76*, 567–576.
16. Jones, L., Ratcliff, F., and Baulcombe, D.C. (2001). RNA-directed transcriptional gene silencing in plants can be inherited independently of the RNA trigger and requires Met1 for maintenance. Curr. Biol. *11*, 747–757.
17. Malecová, B., and Morris, K.V. (2010). Transcriptional gene silencing through epigenetic changes mediated by non-coding RNAs. Curr. Opin. Mol. Ther. *12*, 214–222.
18. Sibley, C.R., Seow, Y., and Wood, M.J. (2010). Novel RNA-based strategies for therapeutic gene silencing. Mol. Ther. *18*, 466–476.
19. Ahlenstiel, C., Mendez, C., Lim, S.T., Marks, K., Turville, S., Cooper, D.A., Kelleher, A.D., and Suzuki, K. (2015). Novel RNA duplex locks HIV-1 in a latent state via chromatin-mediated transcriptional silencing. Mol. Ther. Nucleic Acids *4*, e261.
20. Yamagishi, M., Ishida, T., Miyake, A., Cooper, D.A., Kelleher, A.D., Suzuki, K., and Watanabe, T. (2009). Retroviral delivery of promoter-targeted shRNA induces long-term silencing of HIV-1 transcription. Microbes Infect. *11*, 500–508.
21. Weinberg, M.S., Villeneuve, L.M., Ehsani, A., Amarzguioui, M., Aagaard, L., Chen, Z.X., Riggs, A.D., Rossi, J.J., and Morris, K.V. (2006). The antisense strand of small interfering RNAs directs histone methylation and transcriptional gene silencing in human cells. RNA *12*, 256–262.
22. Kim, D.H., Villeneuve, L.M., Morris, K.V., and Rossi, J.J. (2006). Argonaute-1 directs siRNA-mediated transcriptional gene silencing in human cells. Nat. Struct. Mol. Biol. *13*, 793–797.
23. Turner, A.M., Ackley, A.M., Matrone, M.A., and Morris, K.V. (2012). Characterization of an HIV-targeted transcriptional gene-silencing RNA in primary cells. Hum. Gene Ther. *23*, 473–483.
24. Wong, J.K. (1997). Recovery of replication-competent HIV despite prolonged suppression of plasma viremia. Science *278*, 1291–1295.

25. Hornung, V., Guenthner-Biller, M., Bourquin, C., Ablasser, A., Schlee, M., Uematsu, S., Noronha, A., Manoharan, M., Akira, S., de Fougerolles, A., et al. (2005). Sequence-specific potent induction of IFN-alpha by short interfering RNA in plasmacytoid dendritic cells through TLR7. Nat. Med. *11*, 263–270.

26. Weinberg, M.S., Barichievy, S., Schaffer, L., Han, J., and Morris, K.V. (2007). An RNA targeted to the HIV-1 LTR promoter modulates indiscriminate off-target gene activation. Nucleic Acids Res. *35*, 7303–7312.

27. Jackson, A.L., Bartz, S.R., Schelter, J., Kobayashi, S.V., Burchard, J., Mao, M., Li, B., Cavet, G., and Linsley, P.S. (2003). Expression profiling reveals off-target gene regulation by RNAi. Nat. Biotechnol. *21*, 635–637.

28. Auffray, C., Sieweke, M.H., and Geissmann, F. (2009). Blood monocytes: development, heterogeneity, and relationship with dendritic cells. Annu. Rev. Immunol. *27*, 669–692.

29. Mosser, D.M., and Edwards, J.P. (2008). Exploring the full spectrum of macrophage activation. Nat. Rev. Immunol. *8*, 958–969.

30. Perno, C.F., Svicher, V., Schols, D., Pollicita, M., Balzarini, J., and Aquaro, S. (2006). Therapeutic strategies towards HIV-1 infection in macrophages. Antiviral Res. *71*, 293–300.

31. Verani, A., Gras, G., and Pancino, G. (2005). Macrophages and HIV-1: dangerous liaisons. Mol. Immunol. *42*, 195–212.

32. Donahue, D.A., and Wainberg, M.A. (2013). Cellular and molecular mechanisms involved in the establishment of HIV-1 latency. Retrovirology *10*, 11.

33. Bernard-Stoecklin, S., Gommet, C., Corneau, A.B., Guenounou, S., Torres, C., Dejucq-Rainsford, N., Cosma, A., Dereuddre-Bosquet, N., and Le Grand, R. (2013). Semen CD4+ T cells and macrophages are productively infected at all stages of SIV infection in macaques. PLoS Pathog. *9*, e1003810.

34. Pudney, J., and Anderson, D. (1991). Orchitis and human immunodeficiency virus type 1 infected cells in reproductive tissues from men with the acquired immune deficiency syndrome. Am. J. Pathol. *139*, 149–160.

35. Le Tortorec, A., and Dejucq-Rainsford, N. (2010). HIV infection of the male genital tract–consequences for sexual transmission and reproduction. Int. J. Androl. *33*, e98–e108.

36. Waki, K., and Freed, E.O. (2010). Macrophages and cell-cell spread of HIV-1. Viruses *2*, 1603–1620.

37. Groot, F., Welsch, S., and Sattentau, Q.J. (2008). Efficient HIV-1 transmission from macrophages to T cells across transient virological synapses. Blood *111*, 4660–4663.

38. Thomson, J.A., Itskovitz-Eldor, J., Shapiro, S.S., Waknitz, M.A., Swiergiel, J.J., Marshall, V.S., and Jones, J.M. (1998). Embryonic stem cell lines derived from human blastocysts. Science *282*, 1145–1147.

39. Takahashi, K., Tanabe, K., Ohnuki, M., Narita, M., Ichisaka, T., Tomoda, K., and Yamanaka, S. (2007). Induction of pluripotent stem cells from adult human fibroblasts by defined factors. Cell *131*, 861–872.

40. Yanagimachi, M.D., Niwa, A., Tanaka, T., Honda-Ozaki, F., Nishimoto, S., Murata, Y., Yasumi, T., Ito, J., Tomida, S., Oshima, K., et al. (2013). Robust and highly-efficient differentiation of functional monocytic cells from human pluripotent stem cells under serum- and feeder cell-free conditions. PLoS ONE *8*, e59243.

41. Senju, S., Haruta, M., Matsumura, K., Matsunaga, Y., Fukushima, S., Ikeda, T., Takamatsu, K., Irie, A., and Nishimura, Y. (2011). Generation of dendritic cells and macrophages from human induced pluripotent stem cells aiming at cell therapy. Gene Ther. *18*, 874–883.

42. Gendelman, H.E., Orenstein, J.M., Martin, M.A., Ferrua, C., Mitra, R., Phipps, T., Wahl, L.A., Lane, H.C., Fauci, A.S., Burke, D.S., et al. (1988). Efficient isolation and propagation of human immunodeficiency virus on recombinant colony-stimulating factor 1-treated monocytes. J. Exp. Med. *167*, 1428–1441.

43. Carter, C.A., and Ehrlich, L.S. (2008). Cell biology of HIV-1 infection of macrophages. Annu. Rev. Microbiol. *62*, 425–443.

44. Laguette, N., Sobhian, B., Casartelli, N., Ringeard, M., Chable-Bessia, C., Ségéral, E., Yatim, A., Emiliani, S., Schwartz, O., and Benkirane, M. (2011). SAMHD1 is the dendritic- and myeloid-cell-specific HIV-1 restriction factor counteracted by Vpx. Nature *474*, 654–657.

45. Goldstone, D.C., Ennis-Adeniran, V., Hedden, J.J., Groom, H.C., Rice, G.I., Christodoulou, E., Walker, P.A., Kelly, G., Haire, L.F., Yap, M.W., et al. (2011). HIV-1 restriction factor SAMHD1 is a deoxynucleoside triphosphate triphosphohydrolase. Nature *480*, 379–382.

46. Suzuki, K., Hattori, S., Marks, K., Ahlenstiel, C., Maeda, Y., Ishida, T., Millington, M., Boyd, M., Symonds, G., Cooper, D.A., et al. (2013). Promoter targeting shRNA suppresses HIV-1 infection in vivo through transcriptional gene silencing. Mol. Ther. Nucleic Acids *2*, e137.

47. Suzuki, K., Ishida, T., Yamagishi, M., Ahlenstiel, C., Swaminathan, S., Marks, K., Murray, D., McCartney, E.M., Beard, M.R., Alexander, M., et al. (2011). Transcriptional gene silencing of HIV-1 through promoter targeted RNA is highly specific. RNA Biol. *8*, 1035–1046.

48. Nishimura, T., Kaneko, S., Kawana-Tachikawa, A., Tajima, Y., Goto, H., Zhu, D., Nakayama-Hosoya, K., Iriguchi, S., Uemura, Y., Shimizu, T., et al. (2013). Generation of rejuvenated antigen-specific T cells by reprogramming to pluripotency and redifferentiation. Cell Stem Cell *12*, 114–126.

49. Hockemeyer, D., Soldner, F., Cook, E.G., Gao, Q., Mitalipova, M., and Jaenisch, R. (2008). A drug-inducible system for direct reprogramming of human somatic cells to pluripotency. Cell Stem Cell *3*, 346–353.

50. Methia, N., Louache, F., Vainchenker, W., and Wendling, F. (1993). Oligodeoxynucleotides antisense to the proto-oncogene c-mpl specifically inhibit in vitro megakaryocytopoiesis. Blood *82*, 1395–1401.

51. McAllery, S.A., Ahlenstiel, C.L., Suzuki, K., Symonds, G.P., Kelleher, A.D., and Turville, S.G. (2016). The feasibility of incorporating Vpx into lentiviral gene therapy vectors. Mol. Ther. Methods Clin. Dev. *5*, 16066.

52. Suzuki, K., Craddock, B.P., Okamoto, N., Kano, T., and Steigbigel, R.T. (1993). Poly A-linked colorimetric microtiter plate assay for HIV reverse transcriptase. J. Virol. Methods *44*, 189–198.

53. Suzuki, K., Juelich, T., Lim, H., Ishida, T., Watanebe, T., Cooper, D.A., Rao, S., and Kelleher, A.D. (2008). Closed chromatin architecture is induced by an RNA duplex targeting the HIV-1 promoter region. J. Biol. Chem. *283*, 23353–23363.

54. Suzuki, K., Shijuuku, T., Fukamachi, T., Zaunders, J., Guillemin, G., Cooper, D., and Kelleher, A. (2005). Prolonged transcriptional silencing and CpG methylation induced by siRNAs targeted to the HIV-1 promoter region. J. RNAi Gene Silencing *1*, 66–78.

55. Lim, H.G., Suzuki, K., Cooper, D.A., and Kelleher, A.D. (2008). Promoter-targeted siRNAs induce gene silencing of simian immunodeficiency virus (SIV) infection in vitro. Mol. Ther. *16*, 565–570.

56. Suzuki, K., and Kelleher, A.D. (2009). Transcriptional regulation by promoter targeted RNAs. Curr. Top. Med. Chem. *9*, 1079–1087.

57. Suzuki, K., and Kelleher, A.D. (2010). Lessons from viral latency in T cells: manipulating HIV-1 transcription by siRNA. HIV Therapy *4*, 199–213.

58. Mallardo, M., Dragonetti, E., Baldassarre, F., Ambrosino, C., Scala, G., and Quinto, I. (1996). An NF-kappaB site in the 5′-untranslated leader region of the human immunodeficiency virus type 1 enhances the viral expression in response to NF-kappaB-activating stimuli. J. Biol. Chem. *271*, 20820–20827.

59. Liu, R., Paxton, W.A., Choe, S., Ceradini, D., Martin, S.R., Horuk, R., MacDonald, M.E., Stuhlmann, H., Koup, R.A., and Landau, N.R. (1996). Homozygous defect in HIV-1 coreceptor accounts for resistance of some multiply-exposed individuals to HIV-1 infection. Cell *86*, 367–377.

60. Hütter, G., Nowak, D., Mossner, M., Ganepola, S., Müssig, A., Allers, K., Schneider, T., Hofmann, J., Kücherer, C., Blau, O., et al. (2009). Long-term control of HIV by CCR5 Delta32/Delta32 stem-cell transplantation. N. Engl. J. Med. *360*, 692–698.

61. Anderson, J.S., Walker, J., Nolta, J.A., and Bauer, G. (2009). Specific transduction of HIV-susceptible cells for CCR5 knockdown and resistance to HIV infection: a novel method for targeted gene therapy and intracellular immunization. J. Acquir. Immune Defic. Syndr. *52*, 152–161.

62. DiGiusto, D.L., Krishnan, A., Li, L., Li, H., Li, S., Rao, A., Mi, S., Yam, P., Stinson, S., Kalos, M., et al. (2010). RNA-based gene therapy for HIV with lentiviral vector-modified CD34+ cells in patients undergoing transplantation for AIDS-related lymphoma. Sci. Transl. Med. *2*, 36ra43.

63. Ye, L., Wang, J., Beyer, A.I., Teque, F., Cradick, T.J., Qi, Z., Chang, J.C., Bao, G., Muench, M.O., Yu, J., et al. (2014). Seamless modification of wild-type induced pluripotent stem cells to the natural CCR5Δ32 mutation confers resistance to HIV infection. Proc. Natl. Acad. Sci. USA *111*, 9591–9596.

64. He, J., Chen, Y., Farzan, M., Choe, H., Ohagen, A., Gartner, S., Busciglio, J., Yang, X., Hofmann, W., Newman, W., et al. (1997). CCR3 and CCR5 are co-receptors for HIV-1 infection of microglia. Nature *385*, 645–649.

65. Takayama, N., Nishikii, H., Usui, J., Tsukui, H., Sawaguchi, A., Hiroyama, T., Eto, K., and Nakauchi, H. (2008). Generation of functional platelets from human embryonic stem cells in vitro via ES-sacs, VEGF-promoted structures that concentrate hematopoietic progenitors. Blood *111*, 5298–5306.

66. Trapnell, C., Roberts, A., Goff, L., Pertea, G., Kim, D., Kelley, D.R., Pimentel, H., Salzberg, S.L., Rinn, J.L., and Pachter, L. (2012). Differential gene and transcript expression analysis of RNA-seq experiments with TopHat and Cufflinks. Nat. Protoc. *7*, 562–578.

67. Ramsköld, D., Wang, E.T., Burge, C.B., and Sandberg, R. (2009). An abundance of ubiquitously expressed genes revealed by tissue transcriptome sequence data. PLoS Comput. Biol. *5*, e1000598.

OMTN, Volume *12*

Supplemental Information

Generation of HIV-Resistant Macrophages from IPSCs by Using Transcriptional Gene Silencing and Promoter-Targeted RNA

Kei Higaki, Masako Hirao, Ai Kawana-Tachikawa, Shoichi Iriguchi, Ayako Kumagai, Norihiro Ueda, Wang Bo, Sanae Kamibayashi, Akira Watanabe, Hiromitsu Nakauchi, Kazuo Suzuki, and Shin Kaneko

Figure S1. Karyotype analysis of iPSCs transduced with shRNA.

(a) Karyotyping of sh-transduced iPSC: 95% (19/20) of PromA-transduced and 100% (20/20) of M2-transduced iPSC showed normal karyotype.

(b) X-chromozome FISH assay of PromA-iPSC after sub-cloning: After simple sub-cloning, 99.7% of 1000 PromA-iPSCs showed normal X-choromosomal signal as same as healthy donor PBMC.

Figure S2. PCR analysis of iPSCs transduced with shRNA. (a) Ethidium bromide-stained agarose gels of the products of PCRs performed using shRNA-specific primers, which yielded amplicons of the predicted size in iPSCs transduced with shPromA (PromA-M2) and shPromA-M2 (M2). (b) Ethidium bromide-stained agarose gels of the products of PCRs performed using shRNA-specific primers, which yielded amplicons of the predicted size in macrophages transduced with shPromA (PromA) and shPromA-M2 (M2), but not in macrophages derived from untransduced iPSCs (WT). (c) PCR primer position used for the confirmation of the presence of transgene in the iPSCs.

Figure S3. Flow cytometric analysis of CD4 on iPSC-derived macrophages. Macrophages derived from iPSCs transduced with shPromA or shPromA-M2, and control

iPSCs (PromA, M2, and WT respectively) were analyzed for CD4 expression (black). Unstained macrophages derived from wild-type iPSCs are shown as negative controls (white).

FigureS1

a. Karyotyping of sh-transduced iPSC

G-banding	PromA-iPSC	M2-iPSC
46XX	19/20 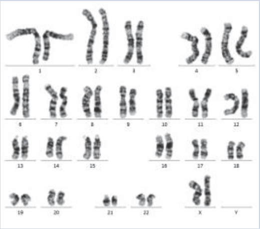	20/20 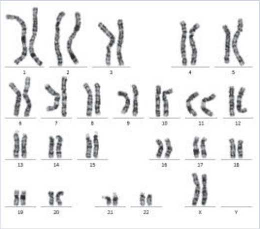
47XXX	1/20 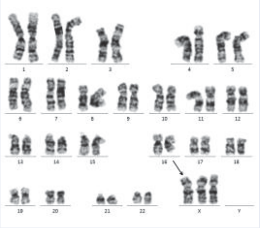	0/20

b. X-chromosome FISH assay of PromA-iPSC after sub-cloning

Signal number by DXZ1(Xcen) probe	PromA-iPSC Cell # (% in 1000 cells)	Healthy donors PBMC Cell # (% in 1000 cells)
1	5 (0.5%)	10 (1.0%)
2	993 (99.3%)	985 (98.5%)
3	2 (0.2%)	5 (0.5%)

FigureS2

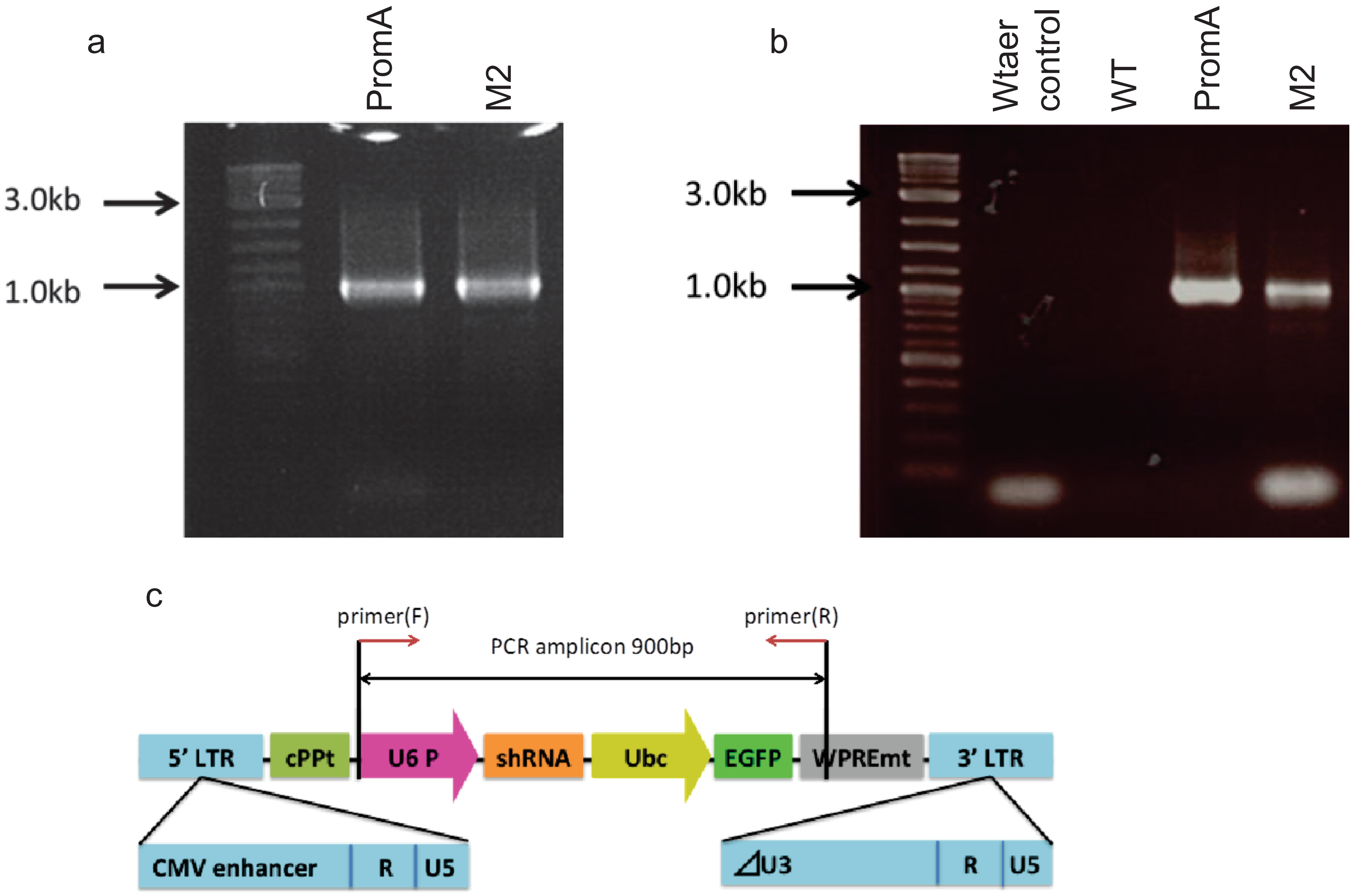

forward primer	5'-CACCGAGGGCCTATTTCCCATGA-3'
reverse primer	5'-ACCTTGTTTCAACGACCTCACAGC-3'

FigureS3

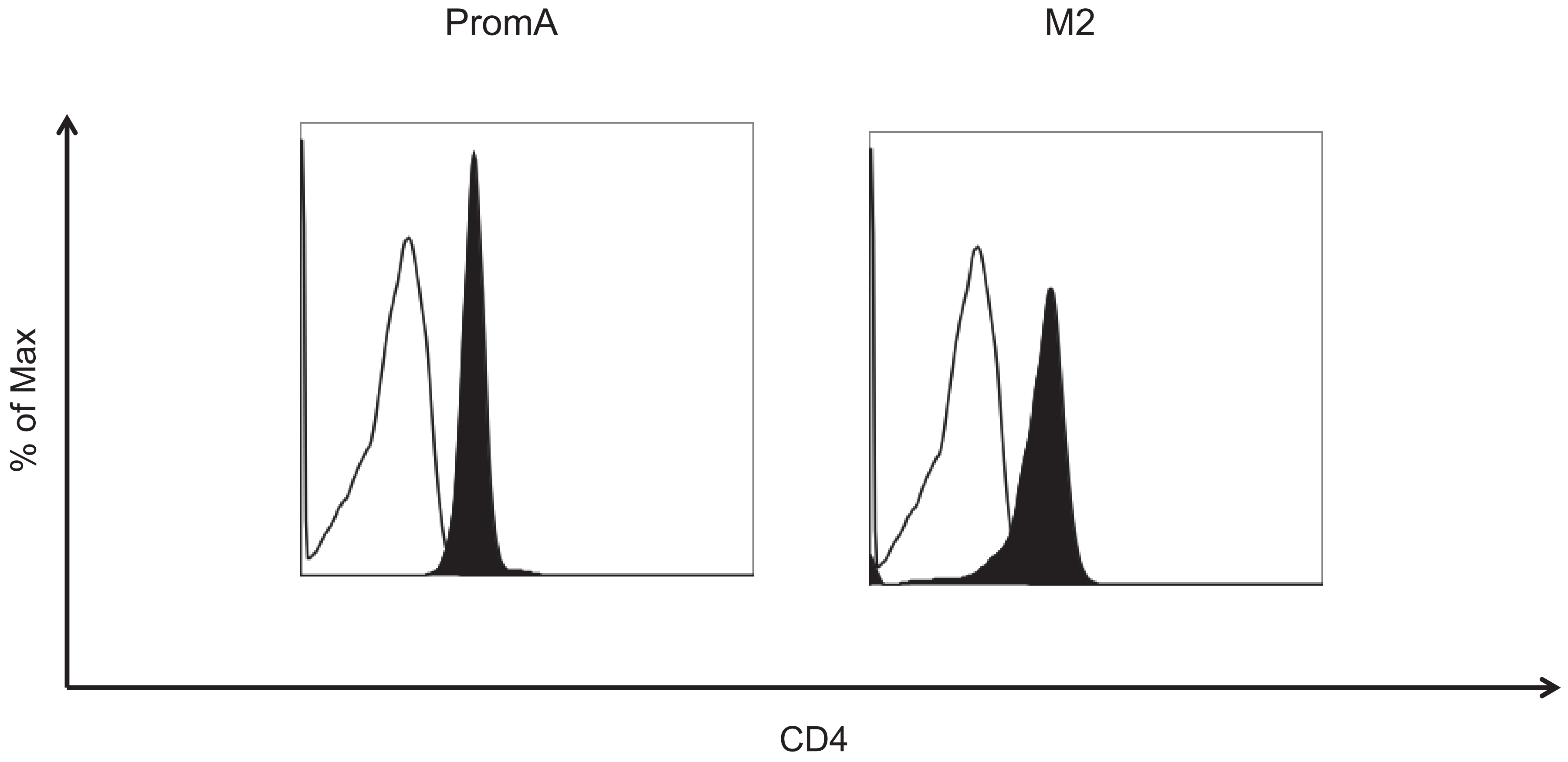

Supplement: Document S2. Article plus Supplemental Information [file mmc3.pdf]
